# Primary Breast Involvement in Erdheim–Chester Disease: A Rare Presentation

**DOI:** 10.1155/crra/8222696

**Published:** 2026-08-31

**Authors:** Kerelus Morkos, Brielle Williams, Ian Simpson, Corinne Ooi, Brooke Sawyer

**Affiliations:** ^1^ Monash Imaging, Monash Health, Clayton, Australia, monashhealth.org; ^2^ Breast Surgery, Monash Health, Moorabbin, Australia, monashhealth.org; ^3^ Monash Pathology, Monash Health, Clayton, Australia, monashhealth.org

**Keywords:** breast, breast neoplasms, Erdheim–Chester disease, mammography

## Abstract

Erdheim–Chester disease (ECD) is a rare multisystem histiocytic neoplasm. Breast manifestations of this condition are exceedingly rare with fewer than 20 cases reported in the literature. We present a case report of a 40‐year‐old female patient with a 5‐year history of breast enlargement and pigmentation who was diagnosed with ECD involving the bones, retroperitoneum and breast, highlighting key imaging and pathologic features that led to the diagnosis. ECD should be considered in the differential diagnosis of infiltrative breast disease. Considering this differential diagnosis allows appropriate further assessment and management.

## 1. Case Report

**Figure 1:**
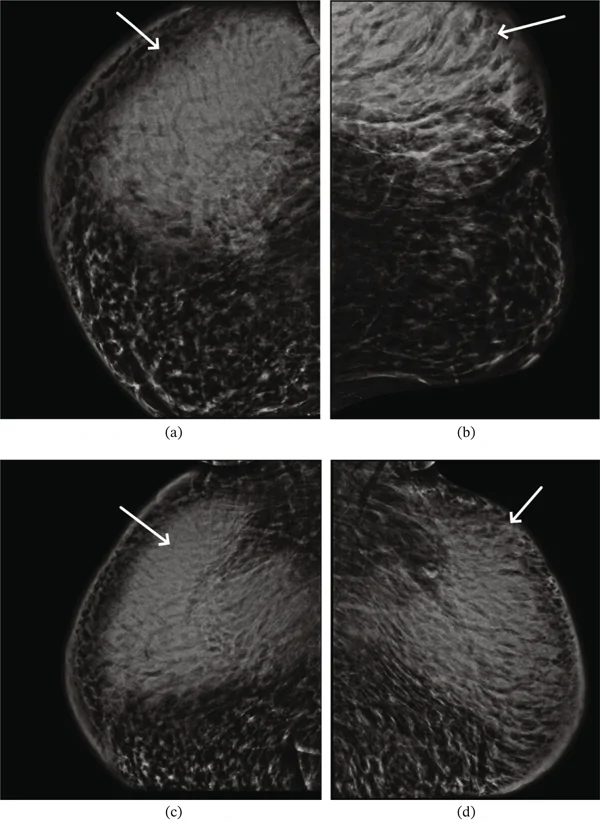
Bilateral MLO and CC digital mammography views. (a) and (b) CC views and (c) and (d) MLO views. Diffusely increased densities with trabecular thickening favouring the upper outer quadrants of both breasts (white arrow). Marked diffuse dermal thickening bilaterally.

**Figure 2:**
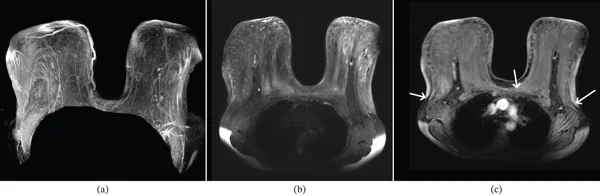
Axial breast MRI images. (a) Maximum intensity projection (MIP) postcontrast image demonstrating symmetric bilateral breast enhancement. (b) STIR image demonstrating diffuse increased T2 signal throughout both breasts. (c) T1 postcontrast fat saturated image highlighting mild diffuse enhancement of the abnormal soft tissue extending to involve the chest wall musculature (white arrow).

**Figure 3:**
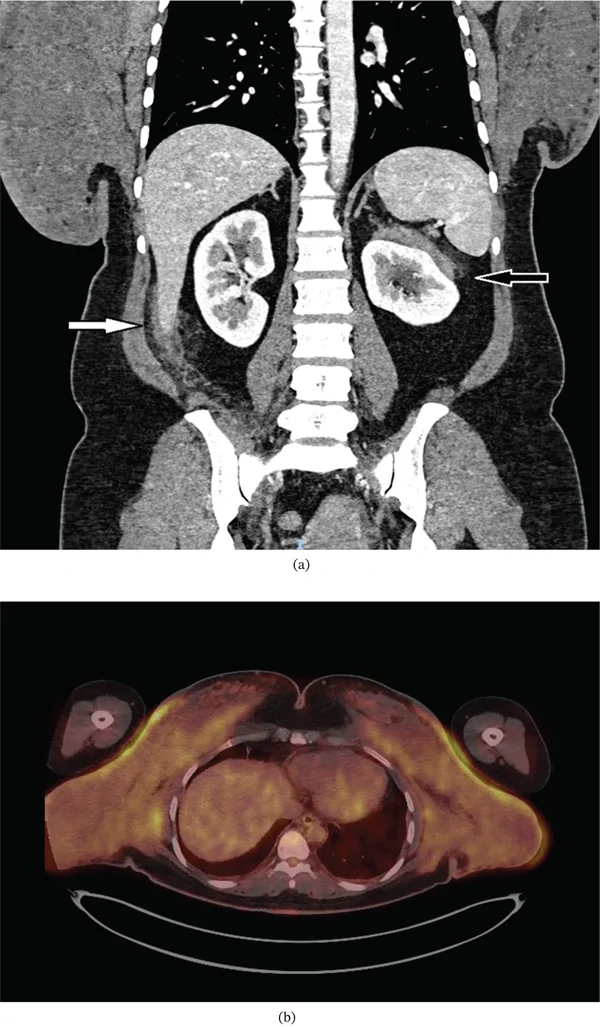
(a) Coronal CT chest, abdomen and pelvis. Bilateral breast and chest wall soft tissue as well as bilateral retroperitoneal (white arrow) and left perinephric soft tissue (black arrow). (b) Fused axial FDG‐PET CT image. Diffuse moderate to intense FDG uptake in the soft‐tissue mass replacing most of the breasts as well as the dermis.

**Figure 4:**
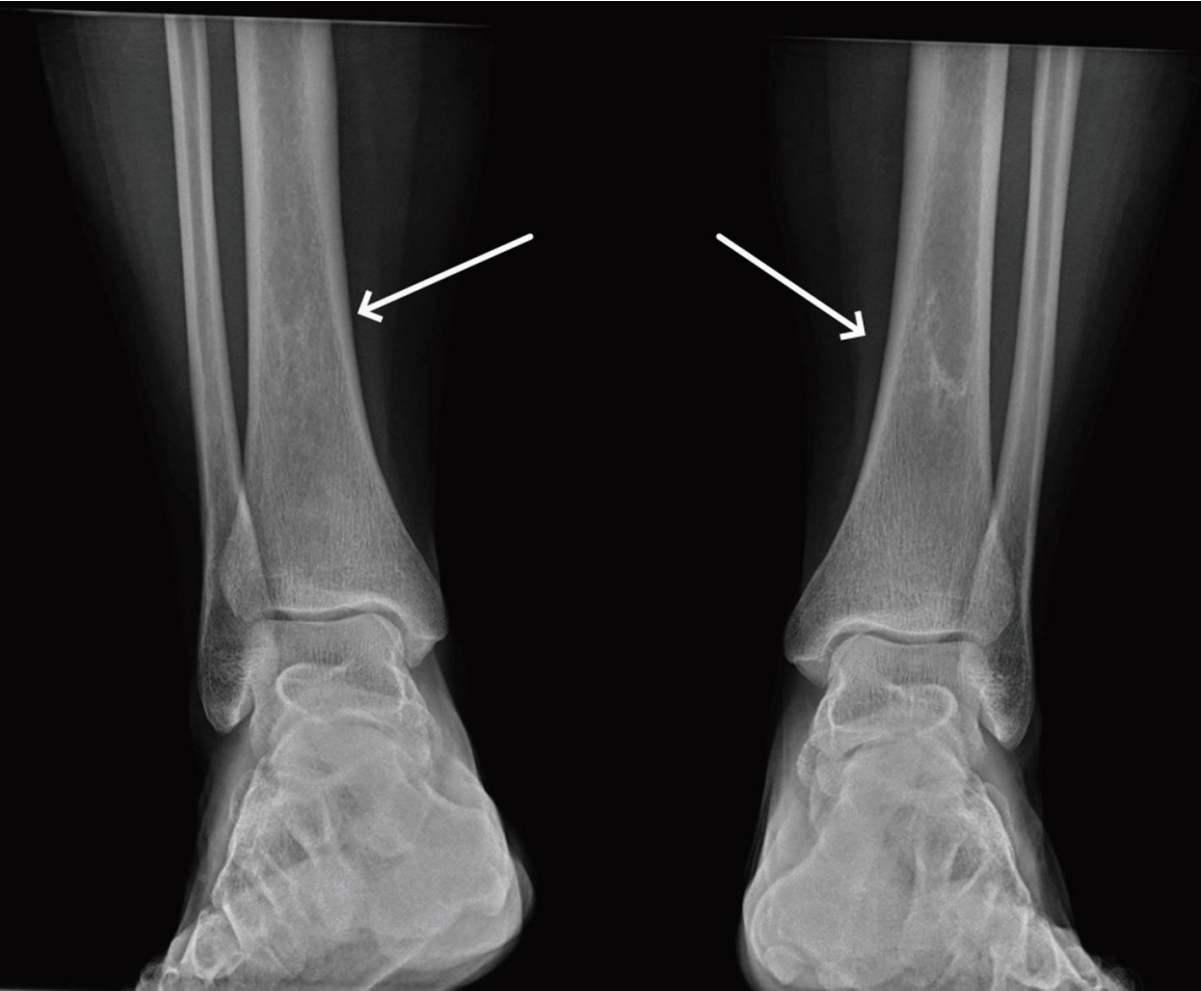
Bilateral frontal ankle radiographs. Characteristic sclerotic osseous lesions of ECD, more conspicuous in the left distal tibia (white arrow).

**Figure 5:**
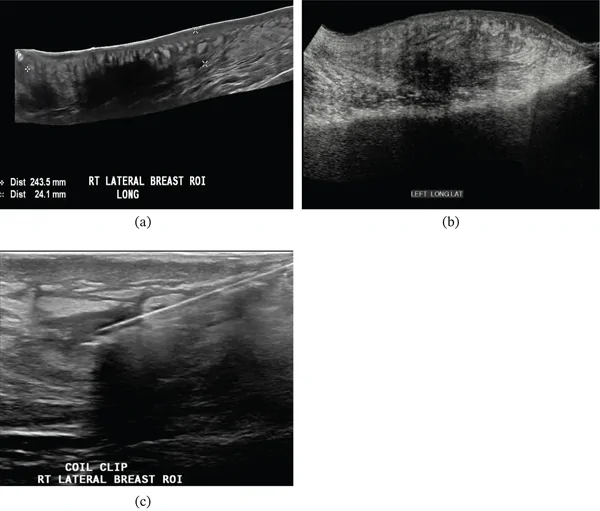
Bilateral breast ultrasound and targeted right lateral breast ultrasound guided core biopsy. (a) Right breast ultrasound demonstrating poorly defined hypoechoic mass with diffuse dermal thickening. (b) Similar appearance in the left breast. (c) Needle position within region of abnormality on ultrasound‐guided core biopsy.

**Figure 6:**
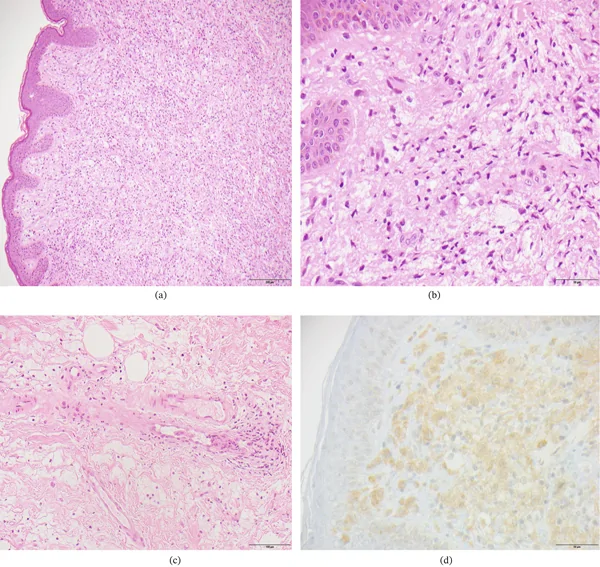
Histopathologic specimen. (a) ×100 H&E slide from breast punch biopsy: sheet‐like proliferation of histiocytes spanning full dermal thickness. (b) ×400 H&E slide from breast punch biopsy: histiocytic proliferation with foamy macrophages and multinucleate giant cells. (c) ×200 H&E slide from breast core biopsy: groups of histiocytes with abundant foamy cytoplasm in a fibrous stroma. (d) BRAF immunoperoxidase on skin specimen: demonstrating diffusely positive staining.

A 40‐year‐old female was referred to the breast surgical clinic with a 5‐year history of bilateral breast enlargement and discolouration. No prior medical history or significant additional complaint was reported. A punch biopsy performed in the clinic showed florid xanthogranulomatous inflammation and lymphohistiocytic cells. Immunohistochemical staining was positive for CD68 and negative for S100 and CD1a. Bilateral mammography and breast ultrasound demonstrated dermal thickening and diffuse soft‐tissue thickening extending to the pectoral muscles bilaterally (Figure 1). Breast MRI highlighted extensive soft‐tissue infiltration displacing the breast tissue centrally and extending laterally to involve the chest wall musculature (Figure 2). In combination with the punch biopsy findings, the possibility of Erdheim–Chester disease (ECD) was raised, and full‐body CT imaging and upper and lower limb skeletal radiography were performed. Bilateral retroperitoneal soft‐tissue infiltration with a ‘hairy kidney’ sign involving the left kidney was observed on CT (Figure 3). Bilateral distal tibial metaphyseal sclerotic lesions were present on the radiographs (Figure 4). An ultrasound‐guided core biopsy of the soft‐tissue infiltrate performed by a radiologist (Figure 5) yielded a histiocytic infiltrate, which was positive for CD68 and Factor VIIIa and negative for CD1a and S100. Additional BRAF V600 molecular testing was weakly positive (Figure 6).

**Figure 7:**
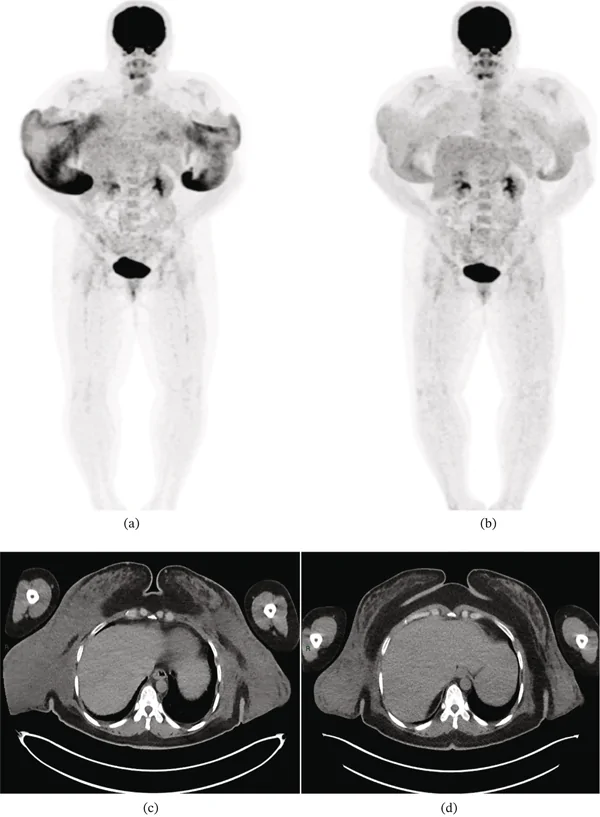
Comparison between index FDG‐PET and 5 months post‐treatment demonstrating partial metabolic and structural response in breast and chest wall lesions. (a) 5 months post‐treatment MIP FDG‐PET. (b) Index MIP FDG‐PET. (c) 5 months post‐treatment axial CT representative image. (d) Index axial CT representative image.

The patient was referred to the haematology service with a presumptive diagnosis of ECD and underwent FDG‐PET imaging which confirmed disease activity in the breasts and retroperitoneum, with no lymphadenopathy or disease activity elsewhere (Figure 3). The patient commenced vemurafenib with clinical reduction in breast masses and discolouration at 6 weeks. Follow‐up FDG‐PET at 5 months post‐treatment initiation confirmed a partial metabolic and structural response (Figure 7).

## 2. Discussion

ECD is a rare histiocytic neoplasm, with 1500 reported cases in the literature (1). Common sites of involvement include the bones, retroperitoneum, orbits and central nervous system. Breast involvement is rare, with fewer than 20 cases described (2). Various patterns of breast involvement, ranging from gynaecomastia in males to focal nodular breast masses, have been reported (3). Our case report describes a case of longstanding breast enlargement and discolouration as a primary presentation of ECD, which appears to be extremely rare. Interestingly, our patient exhibited evidence of osseous and retroperitoneal disease which was clinically asymptomatic at the time of presentation with breast disease. Positive immunohistochemistry for CD68 and molecular testing for BRAF V600 mutations are useful in making the diagnosis and guiding targeted management (4). CD1a negativity helps exclude Langerhans cell histiocytosis (5). Management of ECD has previously included corticosteroids and chemoradiotherapy, with limited efficacy (6). Recent advances, including BRAF inhibitors such as vemurafenib, have shown effectiveness (7). Although limited by a small number of reported cases, 5‐year survival has been reported to be 82.7% (8).

**Learning Points**

• ECD manifesting as diffuse breast disease is rare.
• Recognition of ECD in the breast allows for early detection of a multisystem histiocytic neoplasm.
• ECD should be considered in the differential diagnosis of breast disease, particularly when biopsy yields histiocytic or xanthogranulomatous inflammation.

## Funding

No funding was received for this manuscript.

## Ethics Statement

Human data were utilised for this paper. Written informed consent was obtained from the individual prior to data collection as per institutional protocol.

## Conflicts of Interest

The authors declare no conflicts of interest.

## Supporting Information

Additional supporting information can be found online in the Supporting Information section.

## Supporting information


**Supporting Information 1** Timeline of Patient Care.

**Supporting Information 2** CARE Checklist.

## Data Availability

The data that support the findings of this study are available from the corresponding author upon reasonable request.
